# Mechanisms Underlying the Anti-Inflammatory Effects of *Clinacanthus nutans* Lindau Extracts: Inhibition of Cytokine Production and Toll-Like Receptor-4 Activation

**DOI:** 10.3389/fphar.2016.00007

**Published:** 2016-02-02

**Authors:** Chun W. Mai, Kok S. I. Yap, Mee T. Kho, Nor H. Ismail, Khatijah Yusoff, Khozirah Shaari, Swee Y. Chin, Erin S. H. Lim

**Affiliations:** ^1^Department of Pharmaceutical Chemistry, School of Pharmacy, International Medical UniversityBukit Jalil, Malaysia; ^2^Department of Life Sciences, School of Pharmacy, International Medical UniversityBukit Jalil, Malaysia; ^3^School of Postgraduate Studies and Research, International Medical UniversityBukit Jalil, Malaysia; ^4^Atta-ur-Rahman Institute for Natural Products Discovery, Universiti Teknologi MARAShah Alam, Malaysia; ^5^Institute of Bioscience, Universiti Putra MalaysiaSerdang, Malaysia; ^6^Department of Microbiology, Faculty of Biotechnology and Biomolecular Sciences, Universiti Putra MalaysiaSerdang, Malaysia; ^7^Perdana University-Royal College of Surgeons Ireland, Perdana UniversitySerdang, Malaysia

**Keywords:** anti-inflammatory agents, *Clinacanthus nutans*, Toll-like receptor 4, macrophages, total flavonoid content

## Abstract

*Clinacanthus nutans* has had a long history of use in folk medicine in Malaysia and Southeast Asia; mostly in the relief of inflammatory conditions. In this study, we investigated the effects of different extracts of *C. nutans* upon lipopolysaccharide (LPS) induced inflammation in order to identify its mechanism of action. Extracts of leaves and stem bark of *C. nutans* were prepared using polar and non-polar solvents to produce four extracts, namely polar leaf extract (LP), non-polar leaf extract (LN), polar stem extract (SP), and non-polar stem extracts (SN). The extracts were standardized by determining its total phenolic and total flavonoid contents. Its anti-inflammatory effects were assessed on LPS induced nitrite release in RAW264.7 macrophages and Toll-like receptor (TLR-4) activation in TLR-4 transfected human embryonic kidney cells (HEK-Blue^TM^-hTLR4 cells). The levels of inflammatory cytokines (TNF-α, IFN-γ, IL-1β, IL-6, IL-12p40, and IL-17) in treated RAW264.7 macrophages were quantified to verify its anti-inflammatory effects. Western blotting was used to investigate the effect of the most potent extract (LP) on TLR-4 related inflammatory proteins (p65, p38, ERK, JNK, IRF3) in RAW264.7 macrophages. All four extracts produced a significant, concentration-dependent reduction in LPS-stimulated nitric oxide, LPS-induced TLR-4 activation in HEK-Blue^TM^-hTLR4 cells and LPS-stimulated cytokines production in RAW264.7 macrophages. The most potent extract, LP, also inhibited all LPS-induced TLR-4 inflammatory proteins. These results provide a basis for understanding the mechanisms underlying the previously demonstrated anti-inflammatory activity of *C. nutans* extracts.

## Introduction

*Clinacanthus nutans* (‘*Belalai Gajah*’ in Malay, or ‘*You Dun Cao*’ in Mandarin) has long been used in Thailand to improve bladder function and has also been used traditionally in Malaysia as a folk medicine for kidney and bladder disease (Low et al., 2011). *C. nutans* Lindau (Acanthaceae) leaves and branches have been known to relief nettle rash, dysentery, fever, burns, scalds, insect stings, and oral inflammatory symptoms (Cheeptham and Towers, 2002). Traditionally, *C. nutans* have been consumed as crude extract of the leaves or stem bark. The leaf extracts of *C. nutans* has been reported to possess antioxidant (Khoo et al., 2015), analgesic and anti-inflammatory activities against varicella zoster virus (Thawaranantha et al., 1992) as well as inhibitory activity against scorpion venom-induced fibroblast lysis (Uawonggul et al., 2006). Kittisiripornkul (1984) reported the anti-inflammatory activity of a *n*-butanol-soluble fraction from the leaves (Kittisiripornkul, 1984) and a methanol extract of the whole plant reduced carrageenan-induced paw oedema and ethyl phenylpropiolate-induced ear oedema in rats (Wanikiat et al., 2008). The extract also concentration-dependently inhibited human neutrophil chemokinesis, as well as *N*-formyl-methionyl-leucyl-phenylalanine-induced chemotaxis, superoxide anion generation, and myeloperoxidase and elastase release (Wanikiat et al., 2008). However, the exact cellular mechanisms underlying the anti-inflammatory actions of *C. nutans* extracts remain unknown.

In a recent study (Khoo et al., 2015), it has been highlighted that both polar and non-polar compounds in *C. nutans* could play important roles through its medicinal properties; therefore in our study, we have included both the polar and non-polar fractions of *C. nutans* leaves and stem bark. In order to examine the anti-inflammatory effects of *C. nutans*, we investigated its effects on human embryonic kidney (HEK) cells stably transfected with human TLR (HEK-Blue^TM^-4) and in murine macrophages (RAW264.7) challenged with LPS, the Gram-negative bacterial cell wall component, a well-established activator of TLR-4 (Mai et al., 2013). TLR-4 is the first line of host defense against acute and chronic inflammation and is one of the key pro-inflammatory signaling receptors (Park et al., 2009). Activation of TLR-4 by LPS enhances the production of NO and inflammatory cytokines, through activating nuclear factor κB (NF κB) and IRF3. Inhibition of TLR-4 activation may produce potent anti-inflammatory effects since TLR-4 is the upstream receptor that activates both NF κB and IRF3 signaling, the hallmarks of inflammation (Hatziieremia et al., 2006; Mai et al., 2013).

## Materials and Methods

### Reagents

All materials, unless specified, were purchased from Sigma–Aldrich (St Louis, MO, USA). Ultrapure LPS from *Escherichia coli* was purchased from InvivoGen (San Diego, CA, USA) and was reconstituted using endotoxin-free water (InvivoGen, San Diego, CA, USA).

### Preparation of Plant Extracts

*Clinacanthus nutans* was collected from an orchard in Temerloh, Pahang Malaysia, and its identity was kindly verified by Dr. Richard Chung at the Forest Research Institute of Malaysia. The plant was also deposited in the Malaysian Agricultural Research and Development Institute herbarium with the specimen numbers MDI 12807 and MDI 12808. The plant was separated into leaves and stem bark, which were dried and powdered. The powdered leaves were extracted with polar solvents (methanol and dichloromethane) or non-polar solvents (hexane and diethyl ether) through immersion in the solvent for three days at room temperature. A total of four extracts were prepared, namely polar leave extracts (LP), non-polar leave extracts (LN), polar stem extracts (SP), and non-polar stem extracts (SN). The extracts were then gravity filtered and the solvents removed under vacuum using a rotary evaporator at 60°C. The dried extracts were subjected to further experiments. In order to standardize the extracts, we determined the total phenolic content (TPC) and total flavonoid content (TFC) of extracts using standardized assays as per described in previous studies (Mai et al., 2009a,b). TPC of the extracts were expressed as gallic acid equivalents in milligram per gram of dried material (mg GAE/g dm) while TFC of the extracts were expressed as quercetin equivalent in milligram per gram of dried material (mg QE/g dm).

### Cell Lines and Cell Culture

Macrophages RAW264.7 were obtained from the American Type Culture Collection (ATCC, TIB-71^TM^) and maintained in Dulbecco’s Modification of Eagle’s Medium (DMEM) supplemented with 10% inactivated foetal bovine serum (FBS) and 1% penicillin–streptomycin. All the cells were cultured and maintained at 37°C in a 5% carbon dioxide incubator. HEK-Blue^TM^-4 cells, obtained from InvivoGen (San Diego, CA, USA), are HEK that are stably transfected with human Toll-like receptor-4 (hTLR-4), myeloid differentiation factor-2/cluster of differentiation-14 and SEAP reporter gene. The cells were cultured in complete DMEM which contained 4.5 g/L glucose and L-glutamine, 10% heat inactivate FBS, 1% penicillin-streptomycin, Normocin^TM^ (InvivoGen, San Diego, CA, USA) and HEK-Blue^TM^ Selection Medium (InvivoGen, San Diego, CA, USA). All cells were harvested within 20 passages using the cell scraper, without addition of trypsin for further analysis according to the manufacturer’s instruction.

### Cytotoxicity Assay

The MTT cell viability assay was used to access the cytotoxicity of extracts on HEK-Blue^TM^-hTLR4 cells and RAW264.7 cells, as described previously with modification (Mai et al., 2009a,b, 2013, 2014; Tan et al., 2013). Briefly, all extracts (LP, SP, LN, or SN) were reconstituted using DMSO to 100 mg/mL and further diluted to required concentrations (1–100 μg/mL) using ultra-purified sterile water prior to the assays. Cells were treated with various concentrations of extracts or 0.1% DMSO (negative control) for 72 h before the reaction was terminated with the MTT reagent. The absorbance was recorded at a test wavelength of 570 nm and a reference wavelength of 630 nm using the Tecan Infinite F200 plate reader (Männedorf, Switzerland). The mean absorbance for the negative control (0.1% DMSO) was normalized as 100%.

### Griess Assay

In the presence of LPS, NO is generated by inducible NO synthase in macrophages, as a hallmark of inflammation (Gross and Wolin, 1995; Matsuno et al., 1998). The production of NO can be quantified by measuring the level of nitrite production, the stable metabolite of NO as described in the Griess assay (Kim et al., 1995; Paul et al., 1997; Tsai et al., 1999; Shweash et al., 2011). RAW264.7 macrophages were plated at 2 × 10^5^cells/mL in a 12-well plate and treated with or without extracts (LP, SP, LN, or SN) for 1 h followed by stimulation with or without LPS (100 ng/mL) for 18 h. Supernatants (50 μL) were removed and mixed with equal amounts of Griess reagents (Kim et al., 1995; Paul et al., 1997; Tsai et al., 1999; Shweash et al., 2011). The solutions were then left for 10 min at room temperature before measurement on a microplate reader at 540 nm. The IC_50NO_, indicating the concentration at which extracts inhibited 50% of LPS induced NO production was determined.

### TLR-4 Activation Assay

HEK-Blue^TM^ hTLR4 cells were plated at 1 × 10^5^ cells/mL and extracts were added simultaneously. DMSO (0.1%) was added as a control solvent. In the presence of a TLR-4 agonist, such as LPS, the TLR-4 is expected to be activated. Activated TLR-4 induces NF-κB and activator protein-1 (AP-1) activation, under the control of NF-κB/AP-1 promoter, following which, the promoter will then induce secreted embryonic alkaline phosphatase (SEAP) production. The levels of SEAP production were determined using the HEK-Blue^TM^ Detection Medium (InvivoGen, San Diego, CA, USA), a detection medium which changes color into purple or blue in the presence of SEAP after 24 h incubation. An extract will be accepted as a TLR-4 activator if the percentage of TLR-4 activation is more than 100% as compared cells treated with solvent control, 0.1% DMSO in the absence of LPS. Conversely, an extract will be concluded as a TLR-4 inhibitor if the extract can inhibit LPS induced TLR-4 activation. The SEAP levels were quantified using the Tecan Infinite F200 plate reader (Männedorf, Switzerland) at 630nm. The increase in the level of SEAP is directly proportionate to increased NF-κB activation, resulting in higher degree of TLR-4 activation. Finally, the IC_50TLR4_, indicating the concentration in which extracts inhibited 50% of LPS induced TLR-4 activity was determined.

### Cytokine Assay

Macrophages RAW264.7 cells were plated and either non-treated or treated with extracts (LP, SP, LN, SN) for 1 h followed by exposure to LPS for 18 h. Ten cytokines, namely the interleukin IL-1β, IL-2, IL-4, IL-5, IL-6, IL-10, IL-12p40, IL-17, IFN-γ, and tumor necrosis factor-alpha (TNF-α), were assayed in the supernatants using the MILLIPLEX^®^ MAP Mouse Cytokine/Chemokine Magnetic Bead panel (Millipore, Germany) according to the manufacturer’s instructions. For the plate washing steps, a handheld magnet attached to a plate holder was used and the assay was performed on the Luminex 200 multiplex analyser (Austin, USA) using the Luminex Software xPONENT^®^ 3.1 (Austin, USA) for data acquisition. The Median Fluorescent Intensity data using a 5-parameter logistic or spline curve-fitting method was used for calculating cytokine concentrations in samples. The results were normalised with cells treated with 100 ng/mL of LPS.

### Immunoblotting

Protein lysates from the macrophages treated with 0.1% DMSO, LPS (100 ng/mL); the most potent extract (LP 20 μg/mL) with or without LPS (100 ng/mL) were extracted in an ice-cold lysis buffer (1%-NP-40, 1 mM dithiothreitol and protease inhibitors cocktail in phosphate buffer saline, PBS). Proteins (50 μg) were separated by 7.5% SDS-PAGE, and transferred onto polyvinylidene fluoride (PVDF) membranes. The membranes were blocked for non-specific binding for 1 h in 5% BSA diluted with PBST (PBS and Tween-20). The blots were incubated overnight with respective 50 ng/mL primary antibodies, such as phosphorylated p65 (p-p65), p65, phosphorylated p38 mitogen activated protein kinase (p-p38), p38, phosphorylated ERKs 1/2 (p-ERK 1/2), ERK1/2, phosphorylated c-Jun N-terminal kinase 1/2 (p-JNK 1/2), JNK1/2, phosphorylated interferon regulatory factor 3 (p-IRF3), IRF3, and β-actin. The blots were washed with PBST before incubated with respective horseradish peroxidise-conjugated secondary antibody. All antibodies used in this study were obtained from Cell Signalling Technology, USA. The blots were subjected to enhanced luminol-based chemiluminescent reagents.

### Statistical Analysis

All data were reported as mean ± standard deviation from a minimum of three independent experiments. Statistical significance was analysed using one-way analysis of variance (ANOVA) and post testing using Dunnett’s test through SPSS (version 18.0) for Windows. A *p*-value of less than 0.05 (*p* < 0.05) was considered significantly different compared to negative control, treatment with 0.1% DMSO.

## Results

### *Clinacanthus nutans* Extracts are Not Cytotoxic to HEK-Blue^TM^-hTLR4 or RAW 264.7 Cells

**FIGURE 1:**
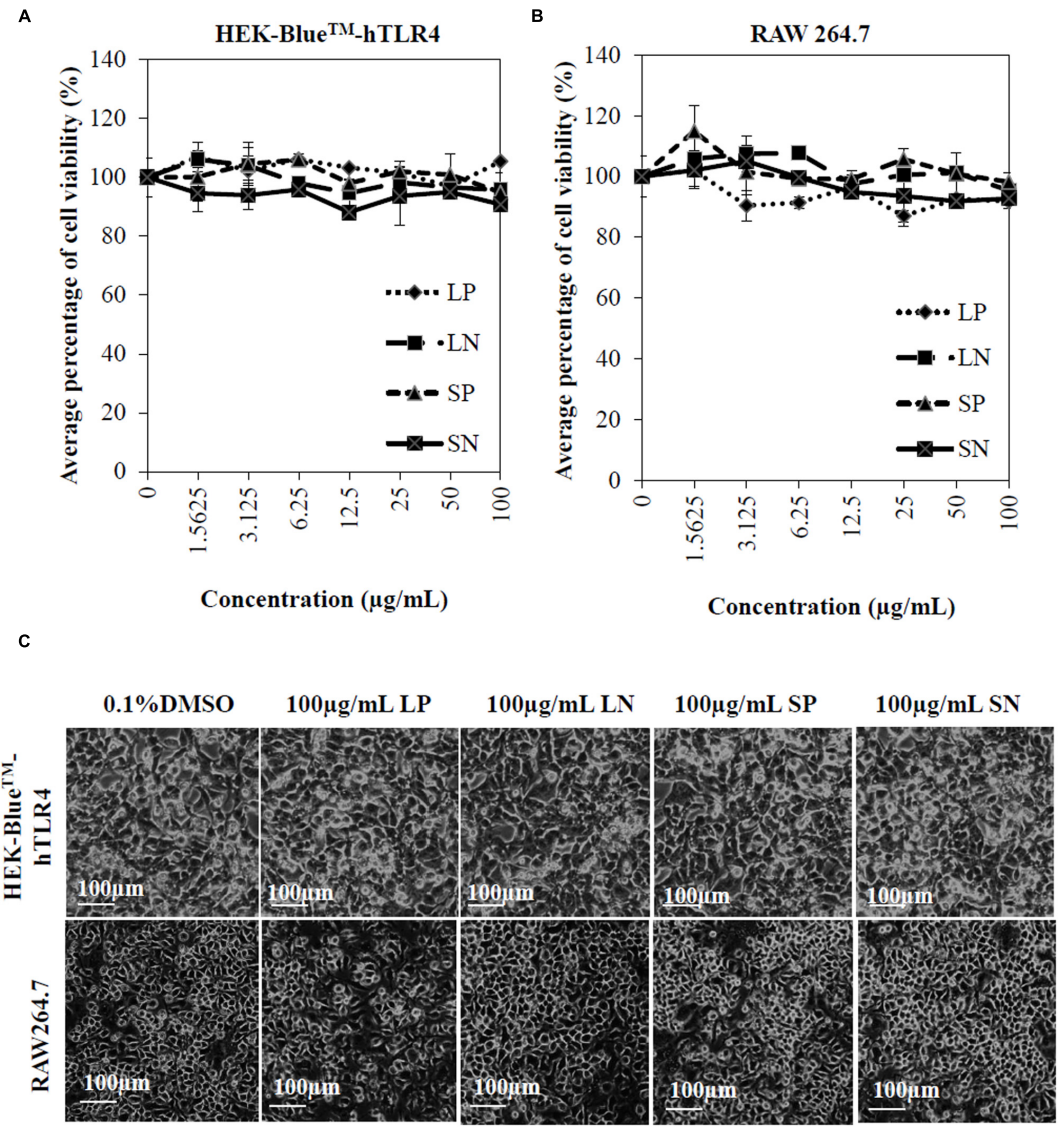
**Cytotoxicity of *Clinacanthus nutans* extracts on cells.** Dose response curve of LP, LN, SP, and SN on cell viability of human embryonic kidney cells, HEK-Blue^TM^hTLR-4 **(A)** and murine macrophages, RAW264.7 **(B)**. All values were not significant difference when compared to 0.1% DMSO (one-way ANOVA *post hoc* Dunnett’s *t*-test). Each value represents means ± SD from three independent experiments. **(C)** No significant morphological changes in HEK-Blue^TM^hTLR-4 and RAW264.7 cells after 72 hours treatment with 100 μg/mL of extracts or 0.1% DMSO.

Cells (HEK-Blue^TM^-hTLR4 cells and macrophages) were treated with various concentrations (1.5625–100 μg/mL) of LP, LN, SP, or SN for 72 h. The viability of HEK cells (HEK-Blue^TM^-hTLR4) and murine macrophages (RAW 264.7) were not significantly reduced by any of the extracts compared to cells treated with 0.1% DMSO (negative control) (**Figures 1A,B**). Microscopic observation (**Figure 1C**) showed no morphological change for all cells treated with 100 μg/mL of the four extracts compared to the negative control cells treated with 0.1% DMSO. The results obtained conclude that there was no cytotoxicity induced by the extracts alone, which could potentially interfere with anti-inflammatory effects.

### *Clinacanthus nutans* Extracts Inhibit LPS Induced NO Production and TLR-4 Activation

**FIGURE 2:**
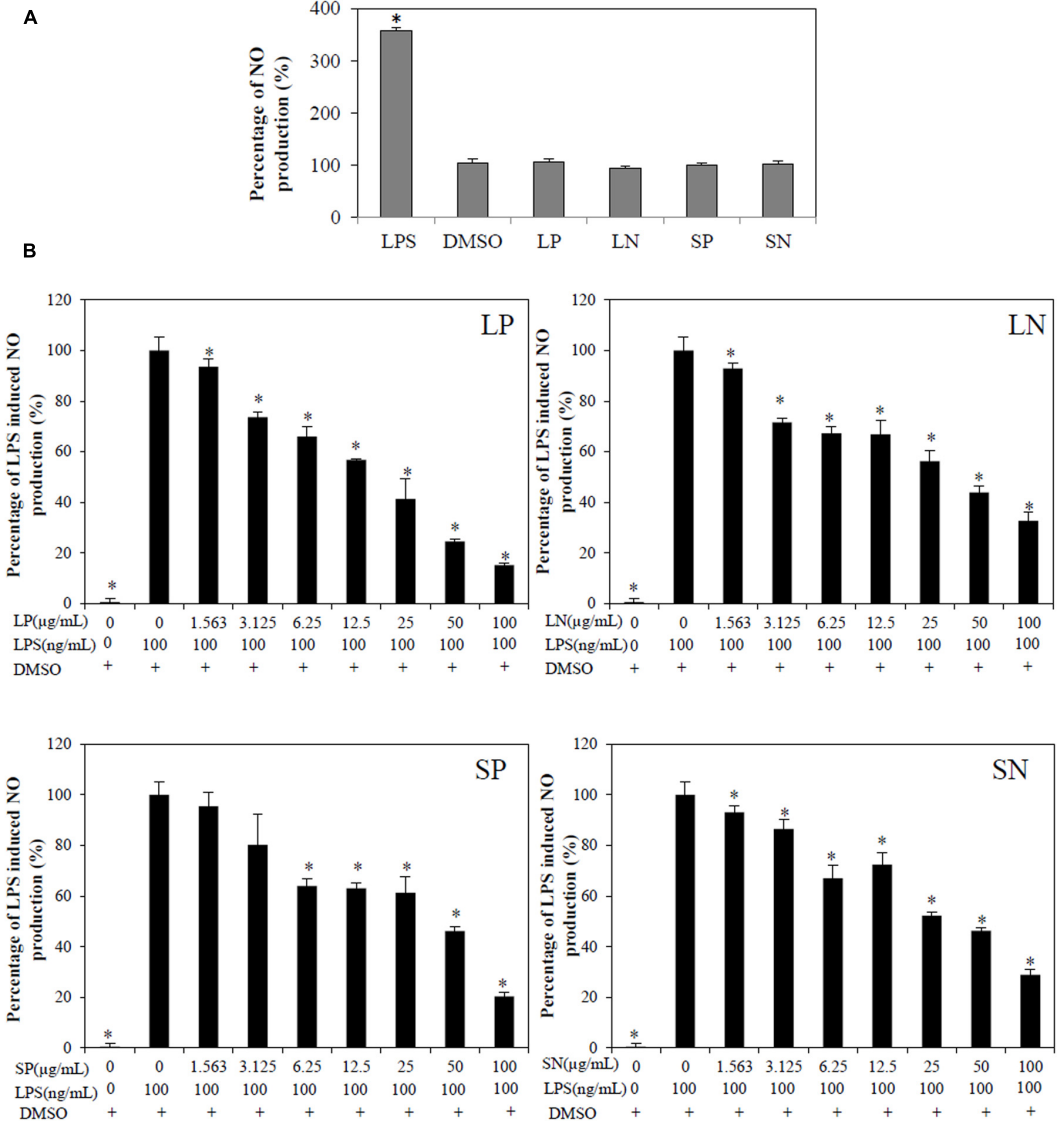
**Effect of *C. nutans* extracts on NO production in RAW264.7 cells. (A)** As compared to cells treated with 0.1% DMSO, cells treated with 100 μg/mL of LP, LN, SP, SN failed to induce significant NO production, while cells treated with 100 ng/mL of LPS induced significant NO production (^∗^*p* < 0.05, one-way ANOVA *post hoc* Dunnett’s *t*-test). **(B)** Cells were treated with various concentrations of extracts (1.5625–100 μg/mL) or 0.1% DMSO for 1 h followed by 18 h of LPS induction (100 ng/mL). Statistical significant difference from LPS induction are indicated as ^∗^*p* < 0.05 by one-way ANOVA *post hoc* Dunnet’s *t*-test. Each value represents means ± SD from three independent experiments.

Lipopolysaccharide induced significant (*p* < 0.05) NO production compared to cells treated with DMSO or with extracts alone (LN, LP, SP, or SN, each 100 μg/mL; **Figure 2A**). All four extracts inhibited LPS induced NO production in a concentration-dependent manner (**Figure 2B**), with LP being the most potent (IC_50NO_= 18.9 ± 3.6 μg/mL; **Table 1**).

**Table 1:** Correlation between TPC and TFC of extracts and its IC_50_ values in Griess (IC_50NO_) and TLR-4 activation assay (IC_50TLR4_).

Extracts	TPC (mg GAE/ g dm)	TFC (mg QE/ g dm)	IC_50NO_ (μg/mL)	Correlation between TPC and IC_50NO_	Correlation between TFC and IC_50NO_	IC_50TLR4_ (μg/mL)	Correlation between TPC and IC_50TLR4_	Correlation between TFC and IC_50TLR4_
LP	7.99 ± 0.6	16.09 ± 4.2	18.9 ± 3.6	*r*^2^ = 0.731	*r*^2^ = 0.839	21.3 ± 5.0	*r*^2^ = 0.764	*r*^2^ = 0.854
LN	3.26 ± 0.9	4.97 ± 1.3	37.1 ± 7.2			29.4 ± 9.0		
SP	2.47 ± 0.4	3.75 ± 0.7	43.1 ± 4.7			27.2 ± 1.0		
SN	1.43 ± 0.1	3.27 ± 1.1	33.8 ± 2.5			27.5 ± 6.3		

*Each value represents means ± standard deviation from three independent experiments*.

**FIGURE 3:**
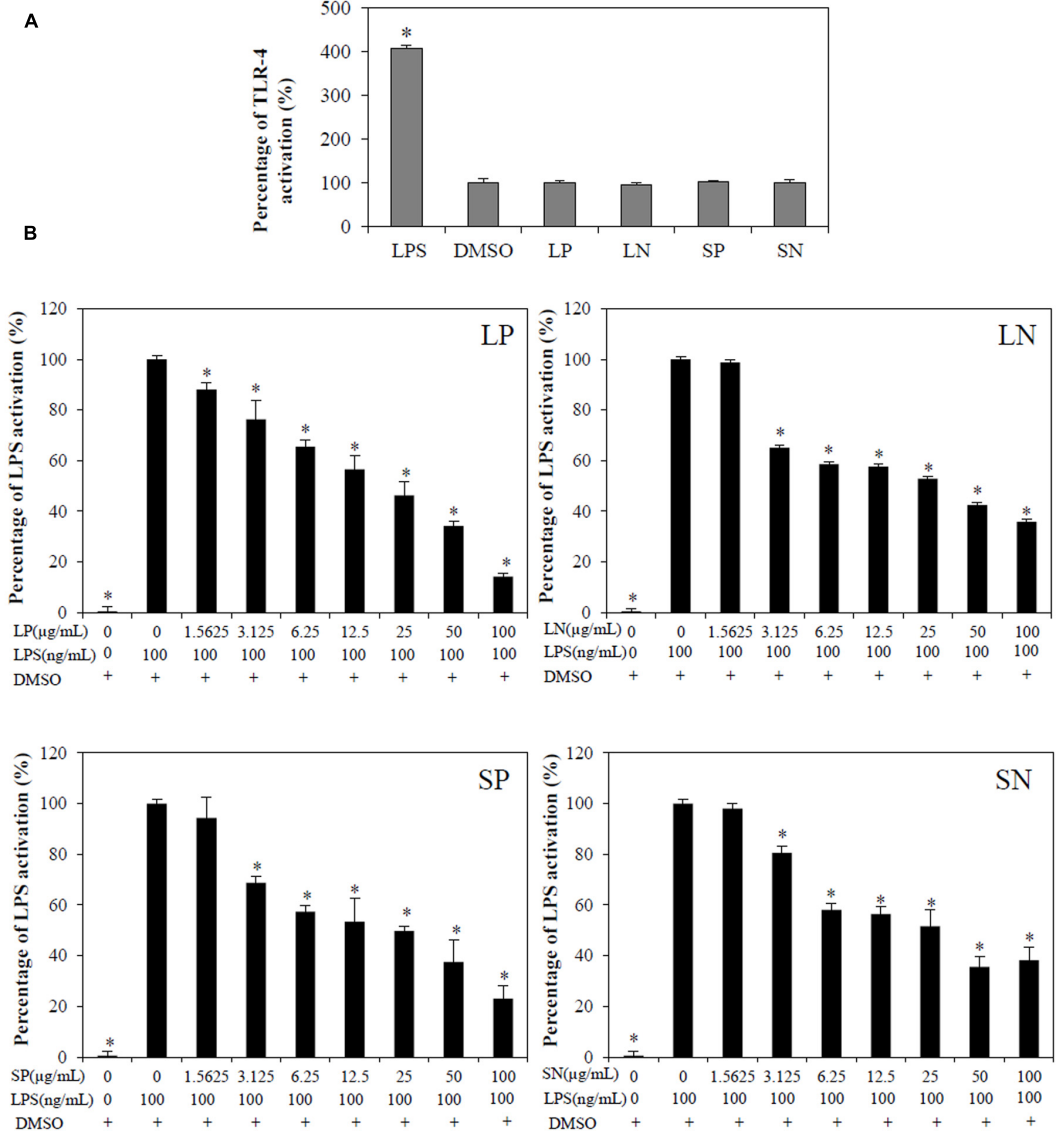
**Effect of *C. nutans* extracts on TLR-4 activation in HEK-Blue^TM^-4 cells. (A)** Cells treated with 100μg/mL of LP, LN, SP, SN failed to induce TLR-4 activation, while 100ng/mL of LPS induced significant TLR-4 activation (^∗^*p* < 0.05, one-way ANOVA *post hoc* Dunnett’s *t*-test) as compared to cells treated with 0.1% DMSO. **(B)** Cells were treated with various concentrations of extracts (1.5625–100 μg/mL) or 0.1% DMSO for 1 h followed by 18 h of LPS induction (100 ng/mL). Statistical significant difference from LPS induction are indicated as ^∗^*p* < 0.05 by one-way ANOVA *post hoc* Dunnet’s *t*-test. Each value represents means ± SD from three independent experiments.

Toll-like receptor-4 activation is the hallmark of inflammation (Mai et al., 2013). LPS (100 ng/mL) induced significant (*p* < 0.05) TLR-4 activation in HEK-Blue^TM^ hTLR4 cells compared with cells treated DMSO or with extracts alone (LN, LP, SP, or SN, each 100 μg/mL; **Figure 3A**). All four extracts inhibited LPS-induced TLR-4 activation in a concentration-dependent manner (**Figure 3B**), with LP being the most potent (IC_50TLR4_= 21.3 ± 5.00 μg/mL; **Table 1**).

### Inhibition of TLR 4 Activation and of NO Production by *C. nutans* Extracts are Related to Its Phenolic Compounds and Flavonoids

*Clinacanthus nutans* extracts indicated content of phenolic compounds (TPC = 1.43 to 7.99 mg GAE/g dm) and flavonoids (TFC = 3.27 to 16.09 mg QE/g dm). The leaf extracts extracted with methanol and dichloromethane (LP), showed the highest TPC and TFC among all the extracts. A higher correlation was obtained between TFC and IC_50NO_ (*r*^2^ = 0.839) compared to correlation between TPC and IC_50NO_ (*r*^2^ = 0.731). Similarly, a higher correlation was obtained between TFC and IC_50TLR4_ (*r*^2^ = 0.854) compared to correlation between TPC and IC_50TLR4_ (*r*^2^ = 0.764). These results suggest the flavonoids in the extracts may play a role in inhibiting LPS-induced NO production and LPS-induced TLR-4 activation.

### *Clinacanthus nutans* Extracts Inhibit LPS Induced Cytokines Production

**FIGURE 4:**
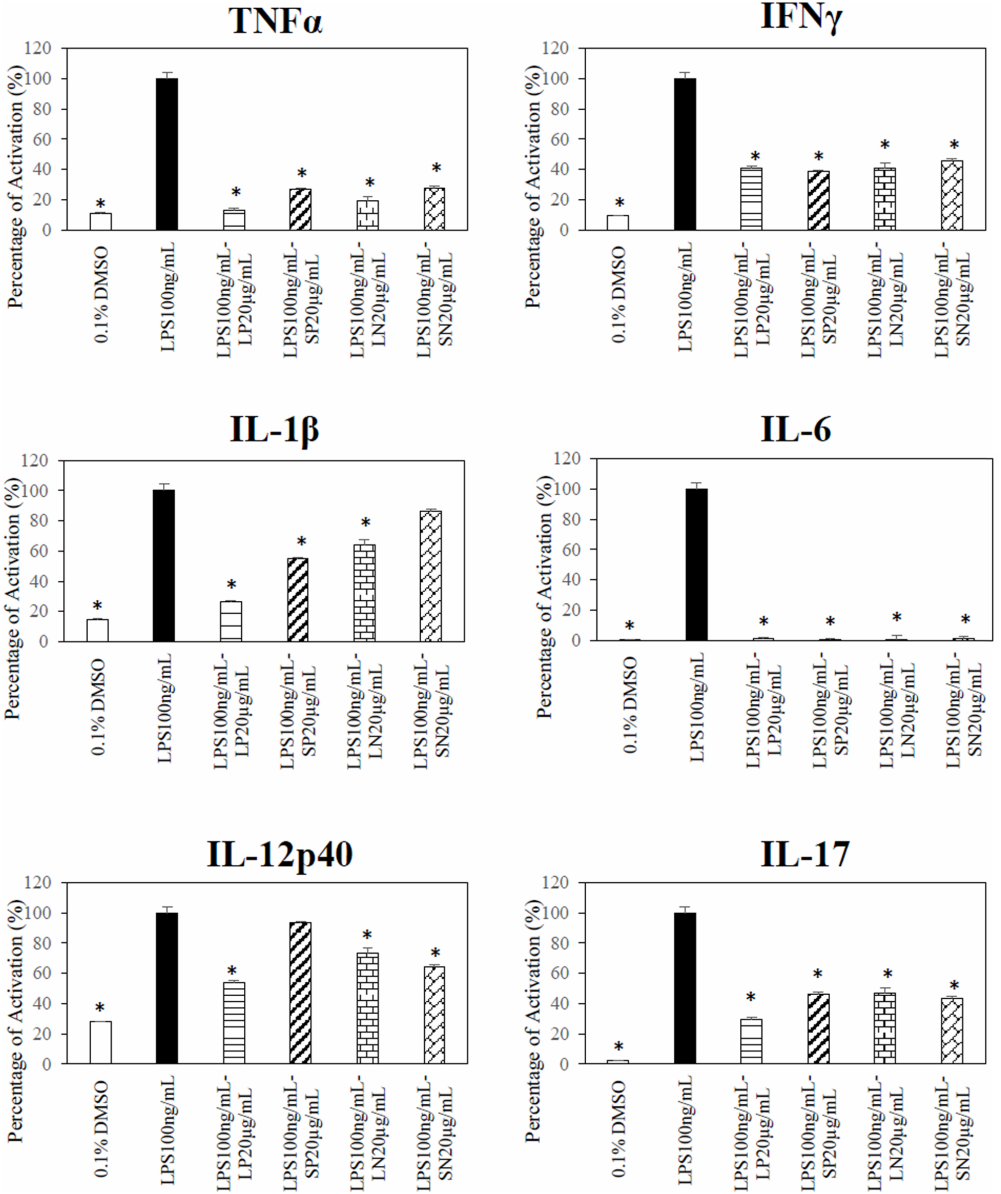
**Effect of *C. nutans* extracts on cytokines in RAW 264.7 cells.** Cells treated with 20 μg/mL of LP, SP, LN, or SN inhibited LPS induce significant cytokines production as compared to cells treated with 100 ng/mL of LPS (^∗^*p* < 0.05, ANOVA *post hoc* Dunnett *t*-test).

Polar leaf extract and LN significantly (*p* < 0.05) inhibited LPS-induced TNF-α, IFN-γ, IL-1β, IL-6, IL12p40, and IL-17 production (**Figure 4**). The SN extract significantly inhibited the LPS-induced production of TNF-α, IFN-γ, IL-6, IL12p40, and IL-17 but not IL-1β, whereas the SP extract inhibited the production of TNF-α, IFN-γ, IL-1β, IL-6, and IL-17 but not that of IL12p40. Four cytokines (IL-2, IL-4, IL-5, and IL-10) were below detectable limits (results not shown).

### *Clinacanthus nutans* extracts inhibit LPS induced TLR-4 inflammatory proteins

**FIGURE 5:**
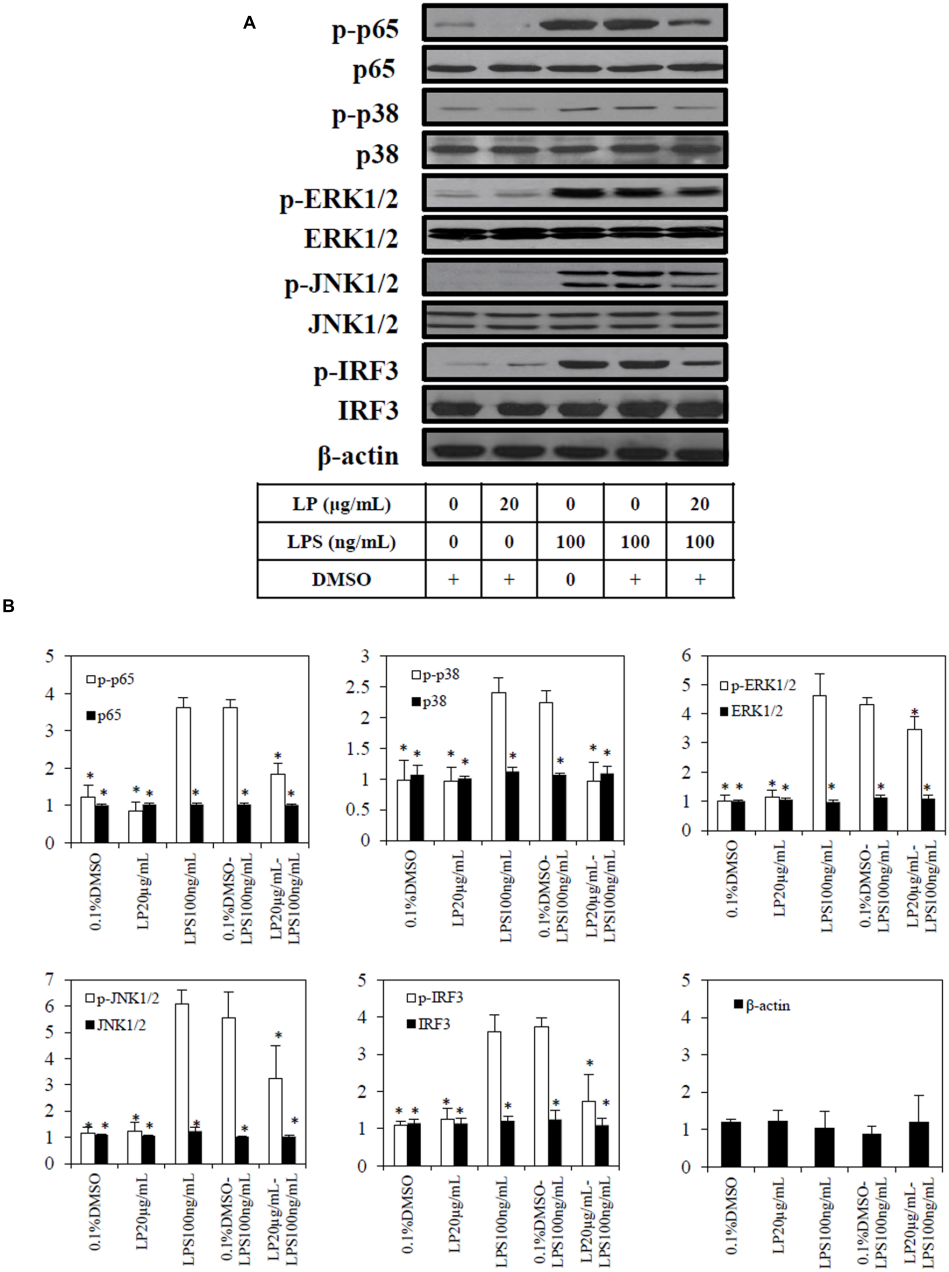
**Effect of extract (LP) on LPS-induced p65, p38, pERK1/2, pJNK 1/2, pIRF3, and β-actin in RAW264.7 macrophages.** RAW264.7 macrophages were pre-treated with vehicle (0.1%DMSO) or the most potent extract (20 μg/mL) of LP for 1 h before stimulation with LPS (100ng/mL) for 18 h. **(A)** Whole-cell extracts were assayed for its phosphorylated and non-phosphorylated p65, p38, pERK1/2, pJNK 1/2, pIRF3, and β-actin as described in Materials and methods. **(B)** Quantification of each blot was performed by scanning densitometry. Each blot is representative of three others. Each value is the mean ± SD of three independent immunoblotting experiments, with ^∗^*p* < 0.05 from macrophages treated with LPS alone.

Based on the results from the Griess assay and TLR-4 activation assay, the most potent anti-inflammatory extract (LP) was selected for immunoblotting. Exposure of macrophages to 100 ng/mL of LPS or combination of 0.1%DMSO and LPS led to phosphorylation of p65, p38, ERK1/2, JNK1/2, and IRF3 (**Figure 5A**)., The extract (20 μg/mL of LP (≈IC_50NO_) significantly (*p* < 0.05) reduced the LPS induced phosphorylation of p65, p38, ERK1/2, JNK1/2, and IRF3. In unstimulated macrophages cells either with LP alone or 0.1% DMSO, there was no significant phosphorylation of p65, p38, ERK1/2, JNK1/2, and IRF3 (**Figure 5B**). No significant change was observed in the loading control proteins, β-actin, between treated and untreated cells.

## Discussion

Although *C. nutans* extracts have been previously shown to reduce superoxide anion production (Tu et al., 2014), and to inhibit neutrophil responsiveness (Wanikiat et al., 2008), the mechanisms underlying its anti-inflammatory effects remain unknown.

Therefore, our study was carried out to examine the effects of extracts on inflammatory processes using the well-established LPS-induced inflammation macrophage model (Paul et al., 1999; Hatziieremia et al., 2006; Cheng et al., 2014; Piwowarski et al., 2015; Zhang et al., 2015). LPS produces a powerful inflammatory response through activation of the TLR-4, resulting in activation of nuclear factor-κB (NF-κB) and the production of NO and inflammatory cytokines, including IFN-γ, TNF-α, IL-1β, IL-6, IL-12p40, and IL-1 (Medzhitov et al., 1997; Kawai and Akira, 2007; Trinchieri and Sher, 2007). In the present study, we found that the extracts of *C. nutans* produced a marked and concentration-dependent inhibition of the production of NO and the inflammatory cytokines; this was clearly unrelated to cytotoxicity caused by the extracts when administered alone. The roles of NO (Hatziieremia et al., 2006; Gomez et al., 2013), IL-1β (Maelfait et al., 2008), TNF-α (Prince et al., 2004; Noman et al., 2009), IFN-γ (Yamada et al., 2005), IL-6 (Greenhill et al., 2011), IL-12 (Krummen et al., 2010), and IL-17 (Derkow et al., 2015) in the TLR-4 related inflammatory responses have been well established. The suppression of the production of NO and these cytokines suggested that the anti-inflammatory effects of these extracts were related to inhibition of TLR-4 activation.

Unlike other TLR, TLR-4 is the only TLR that activates both myeloid differentiation primary response 88 (MyD88) dependent and Toll/IL-1R domain containing adapter inducing IFN-β (TRIF) dependent pathways in the presence of LPS. MyD88 recruits the IL-1 receptor-associated kinases and causes the activation of NF-κB, (such as p65), p38, ERK, and Jun N-terminal kinase (JNK) (Kawai and Akira, 2007). NF-κB activation, a downstream pathway of TLR-4, is a hallmark for inflammation, contributing to chronic diseases such as inflammatory bowel disease, systemic inflammatory response syndrome, and chronic inflammatory demyelinating polyradiculoneuritis (Tak and Firestein, 2001). In the TRIF pathway, TRIF activates tumor necrosis factor receptor-associated factor (TRAF) family member through TRAF3 (Kawai and Akira, 2010). TRAF3 promotes IRF3 activation and subsequently IFN-γ induction (Kawai and Akira, 2010; Mai et al., 2013). The most potent of our extracts (LP) inhibited LPS-induced phosphorylation of p65, p38, ERK, JNK, and IRF3. These results correlated with the ability of the extracts to inhibit LPS induced TLR-4 activities. Moreover, the extract reduced LPS-induced production of IFN-γ and phosphorylation of IRF3. These are the first results showing *C. nutans* to target both the MyD88 dependent and TRIF dependent pathways. The finding that TLR-4 activation was prevented by the extracts of *C. nutans* as measured by SEAP production in response to LPS, lends further support to the hypothesis that *C. nutans* exerts its anti-inflammatory effects by preventing the activation of the TLR-4 receptor, thus reducing the production of inflammatory cytokines and TLR-4 related inflammatory proteins. At which point(s) of this pathway the extract produces its effects remain(s) to be determined.

We found good correlations (*r*^2^ > 0.7) between TPC and TFC with the potency of the extracts in inhibiting NO production (IC_50NO_) and TLR-4 activation (IC_50TLR4_), suggesting both phenolic compounds and flavonoids in the extracts could mediate potential anti-inflammatory effects. The greater correlation between TFC of extracts and its anti-inflammatory effects could be largely attributed to the presence of flavonoids, as compared to phenolic compounds in consideration of its correlation to TPC. These results corroborate with other findings whereby both flavonoids (schaftoside, gendarucin A, apigenin) and phenolic compounds (gallic acid, 3,3-di-*O*-methylellagic acid) were present in *C. nutans* Lindau leave extracts (Khoo et al., 2015). The leaf extracts in the study by Khoo et al. (2015) had similar TPC values to those in the LP extracts in our study although different extraction methods were used. Previous studies indicated that these flavonoids (Erel et al., 2011; Funakoshi-Tago et al., 2011; Zhang et al., 2014) and phenolic compounds(Sgariglia et al., 2013; Pandurangan et al., 2015) identified by Khoo et al. (2015) exerts anti-inflammatory effects. Further studies should be carried out to elucidate the active compounds in leave extracts (LP) and to consolidate the scientific evidence especially the mechanisms involved, in order to further enhance the use of *C. nutans* as an anti-inflammatory agent.

## Conclusion

This study provides evidence that the crude extracts of *C. nutans* leaves exert their anti-inflammatory effects by inhibiting TLR-4 activation. These results provide a basis for understanding the mechanisms underlying the traditionally belief of anti-inflammatory of *C. nutans* crude extracts. More studies using pathway specific inhibitors or a genetic knockout of TLR-4 may further elucidate the mechanism of action of the extracts in specific pathways of TLR-4. A detailed isolation of bioactive compounds from LP extracts would be highly warranted in view of the potential anti-inflammatory attributes of *C. nutans*.

## Author Contributions

Cell culture experiments, inflammation studies, and immunoblotting were conducted by CM. Cytokine assays were designed and conducted by KSY, MK, KY, and EL. Preparation of plant extracts were designed and completed by NI, KS, SC, and EL. All authors contributed toward data analysis, drafting, and revising the manuscript.

## Conflict of Interest Statement

The authors declare that the research was conducted in the absence of any commercial or financial relationships that could be construed as a potential conflict of interest.

## Abbreviations

BSA: Bovine serum albumin
DMSO: Dimethyl sulfoxide
ERK: Extracellular signal-regulated kinase
HEK-Blue^TM^-hTLR4: TLR-4 transfected human embryonic kidney cells
IC_50NO_: Concentration inhibits 50% of LPS induced NO production
IC_50TLR4_: Concentration inhibits 50% of LPS induced TLR-4 activity
IFN-γ: Interferon-gamma
IL: Interleukin
IRF3: Interferon regulatory factor 3
JNK: c-Jun N-terminal kinase ½
LN: Non-polar leaf extract
LP: Polar leaf extract
LPS: Lipopolysaccharide
mg GAE/g dm: Gallic acid equivalent (mg) per dried material (g)
mg QE/g dm: Quercetin equivalent (mg) per dried material (g)
MTT: Methyl thiazolyl tetrazolium
NF κB: Nuclear factor κB
NO: Nitric oxide
SDS-PAGE: Sodium dodecyl sulfate polyacrylamide gel electrophoresis
SEAP: Secreted embryonic alkaline phosphatase
SP: Polar stem extract
SN: Non-polar stem extract
TFC: Total flavonoid content
TLR-4: Toll-like receptor-4
TNF-α: Tumor necrosis factor-alpha
TPC: Total phenolic content
